# SLERT, as a novel biomarker, orchestrates endometrial cancer metastasis via regulation of BDNF/TRKB signaling

**DOI:** 10.1186/s12957-022-02821-w

**Published:** 2023-01-31

**Authors:** Jun Tian, Hailing Cheng, Ning Wang, Chenhui Wang

**Affiliations:** Department of Gynaecology, Huaihe Hospital of Henan University, 1 Baogong Hubei Road, Gulou District, Kaifeng City, 475001 Henan Province China

**Keywords:** Endometrial cancer, SLERT, m^6^A modification, EMT, Biomarker

## Abstract

**Background:**

Recent evidence suggests that the box H/ACA small nucleolar RNA (snoRNA)-ended long noncoding RNA (lncRNA), SLERT, plays a critical role in gene regulation. However, its role in cancer remains undetermined. Herein, we explored its implication in human endometrial cancer (EC).

**Methods:**

EC plasma and tissue samples were collected for the detection of SLERT expression using qRT-PCR method. The functional investigation was tested by CCK-8 and transwell assays. Luciferase reporter, RNA pull-down, and immunoprecipitation (RIP) assays were used to determine the regulatory network involved in SLERT. The in vivo effect of SLERT was tested by caudal vein lung metastasis model.

**Results:**

Stable knockdown of SLERT significantly inhibited EC cell (KLE and AN3CA) migration and invasion, while it did not affect cell viability. SLERT induced epithelial-mesenchymal transition (EMT) via elevating N-cadherin and Vimentin and downregulating E-cadherin. Further investigation showed that SLERT directly binds to METTL3, increasing the m^6^A levels of BDNF mRNA; then, the m^6^A sites were read by IGF2BP1, enhancing BDNF mRNA stability, followed by the activation of BDNF/TRKB signaling, an inducer of EMT. The animal model showed that overexpression of SLERT increased EC cell lung metastasis, and this effect was effectively blocked by BDNF silencing or treatment with TRKB inhibitor k252a. Clinically, EC patients have high levels of SLERT both in tissue or plasma, which might be used as a biomarker of diagnosis and prognosis.

**Conclusion:**

Our findings, for the first time, uncover the metastasis-promoting effect of SLERT in EC via in vitro and in vivo evidence, providing a potential therapeutic target for metastatic EC treatment.

**Supplementary Information:**

The online version contains supplementary material available at 10.1186/s12957-022-02821-w.

## Introduction

Endometrial cancer (EC) is a kind of epithelial malignant tumor occurring in the endometrium, most commonly occurring in perimenopausal and postmenopausal women [1]. It is the sixth most commonly occurring female cancer [2]. Although our understanding of EC biology has recently made significant progress, many aspects of treatment are still controversial, including the role of surgical lymph node evaluation and the choice of patients with adjuvant radiotherapy or chemotherapy [3]. Although 67% of patients present with early-stage disease, which has an 81% 5-year overall survival (OS), the 5-year OS for stage IVA and IVB EC are only 17% and 15%, respectively [4]. Therefore, it is of great significance to deeply explore the potential mechanism of EC metastasis for clinical prevention and treatment.

Long non-coding RNA (lncRNA) is a class of endogenous RNA molecules that do not encode proteins and the transcript length is over 200nt, which can regulate gene expression on multiple levels (epigenetic regulation, transcriptional regulation, and post-transcriptional regulation, etc.) [5]. LncRNA was originally thought to be a byproduct of RNA polymerase II transcription, a “noise” that had no biological function. However, studies in recent years have shown that lncRNA is involved in X chromosome silencing, chromosome modification, genome modification, transcriptional activation or interference, and nuclear transport, and its regulatory role is being extensively studied [6]. The mechanism of action of lncRNA is extremely complex and is not fully understood. Emerging evidence suggests that lncRNA is involved in the progression of human disease as protein binding partners [7]. For instance, lncRNA SNHG11 enhanced HIF-1α protein stability via binding to HIF-1α, because it prevented pVHL-mediated ubiquitination and degradation of HIF-1α [8]. LncRNA GAL is directly bound to GLUT1 and increased GLUT1 SUMOylation, promoting colorectal cancer liver metastasis [9]. LncRNA MSTO2P promoted cancer cell proliferation and invasion via interacting with EZH2, an epigenetic silencer, thus inhibiting CDKN1A expression [10]. These studies highlight that lncRNA acts as the decisive participant in the development of human cancer, rather than meaningless transcriptional garbage.

Recently, a novel lncRNA, SLERT, has been reported to enhance pre-rRNA transcription via evicting DDX21 suppression on Pol I transcription, leading to decreased tumorigenesis [11, 12]. However, whether SLERT functions in EC remains unknown. In this study, we first examined the level of SLERT in EC and found its overexpression. Further, we used a series of in vitro and in vivo assays to clarify the potential mechanism of SLERT promoting EC metastasis.

## Materials and methods

### Tissue, plasma samples, and cell lines

This study included 92 EC tissues and 28 normal tissues, which were collected from Huaihe Hospital of Henan University. The fresh tissues were stored in liquid nitrogen immediately to prevent RNA degradation. In addition, plasma samples were also collected from 40 EC patients and 18 healthy volunteers. Those who had received preoperative chemoradiotherapy were excluded. All procedures are approved by the Medical Institutional Review Board of Huaihe Hospital of Henan University (Approval No: 2020151). Two EC cells KLE and AN3CA were commercially purchased from China Center for Type Culture Collection (CATCC). DMEM medium was used to culture cells at 37°C with 5% CO_2_.

### Quantitative real-time PCR (qRT-PCR) analysis

Total RNA was extracted from EC cells using Trizol reagent (Invitrogen, CA, USA). The Cytoplasmic & Nuclear RNA Purification Kit (Norgen Biotek Corp., B.C, CAN) was used to isolate cytoplasmic and nuclear RNA fragments. Then, reverse transcription was conducted using SuperScript™ reverse transcriptase (Invitrogen) and gene amplification and quantification were tested by PowerUp™ SYBR™ Green (Invitrogen) with the QuantStudio™ 7 Pro system (Invitrogen). GAPDH was used as the reference control for quantifying SLERT and BDNF expression.

### Fluorescent In Situ Hybridization (FISH)

To visualize the location of SLERT in cells, the FISH Kit was purchased (BersinBio, Guangzhou, China) and the assay was conducted as per the manufacturer’s instructions with minor modifications. In brief, the FAM-labeled probe against SLERT was synthesized and incubated with cells permeated with ethanol at 37°C for 4h. After washing for six times, cells were counterstained with DAPI and then observed with fluorescence microscope.

### Lentiviral vector, siRNA, and plasmid

Four shRNAs targeting SLERT were designed and inserted into YSH-LV001-shRNA lentiviral vector (Yuanjing Biotechnology Co, Guangzhou, China), followed by infection into KLE and AN3CA cells in the presence of polybrene. The stable cell lines were screened by 2ug/mL puromycin. The siRNAs targeting IGF2BP1, BDNF, and METTL3 were commercially purchased from Santa Cruz Biotechnology (CA, USA). To overexpress BDNF and SLERT, the full-length sequence was synthesized and inserted into pcDNA 3.0 vector (Invitrogen).

### CCK-8, wound healing, and transwell assays

Cell viability was detected by CCK-8 solution (Dojindo, Kumamoto, Japan) in 96-well plates, followed by assessment of absorbance at 450nm with the automatic enzyme-linked immunosorbent assay system. Cell migration was tested by wound healing assay, the migration distance in each well was recorded at 48h. Transwell chamber coated with matrigel was used to test cell invasion; the invaded cells were stained by crystal violet.

### Western blot

KLE and AN3CA cells with stable SLERT knockdown were lysed by RIPA buffer supplemented with protease inhibitor cocktail (Roche, Basel, CH). Protein quantification was carried out using Pierce™ BCA Protein Quantitative Kit (Invitrogen). Total protein was separated by 10% SDS-PAGE gel and transferred to PVDF membrane. After blocking with 5% skimmed milk, the membrane was incubated with primary antibodies: anti-E-cadherin (#14472, CST), anti-N-cadherin (#13116, CST), anti-Vimentin (#5741, CST), anti-Snail (#13099-1-AP, Proteintech), anti-METTL3 (#86132, CST), anti-BDNF (#47808, CST), anti-TRKB (#4603, CST), and anti-GAPDH (sc-47724, Santa Cruz Biotechnology). Lastly, the PVDF membrane was developed using SuperSignal West Pico PLUS (Invitrogen).

### Luciferase reporter assay

The promoter of BDNF (2000bp) was synthesized and inserted into pGL3-basic vector (Promega, WI, USA), followed by transfection into HEK293T, KLE, and AN3CA cells using Lipofectamine 3000 (Invitrogen) according to the manufacturer’s instructions. The promoter activity was tested by luciferase reporter system (Promega).

### Methylated RNA immunoprecipitation (MeRIP)

Total RNA was extracted from KLE and AN3CA cells using Trizol solution. Then, RNA was purified and fragmented with dynabeads mRNA Purification Kit (Invitrogen) and RNA fragmentation reagent (Invitrogen), respectively. After incubation with anti-m^6^A antibody (ab286164, Abcam), the enrichment of BDNF mRNA was tested by qRT-PCR method.

### RNA pull-down and RIP assays

The biotin-labeled probes against SLERT and its anti-sense were synthesized and incubated with cell lysate at 4°C overnight. After incubation with Magnetic Streptavidin Beads (Invitrogen) at 25°C for 2h, the enriched protein was washed and subjected to western blot assay. For RIP assay, the corresponding antibodies were added into cell lysate, and the enriched RNA was purified and tested by qRT-PCR assay.

### In vivo lung metastasis model

A total of 20 NOD/SCID mice of similar weight were used in this study, which were approved by the Institutional Animal Care and Use Committee of Huaihe Hospital of Henan University. The mice were randomly divided into four groups (*n*=5 per group), labeled as OE-vector (caudal vein injection of 2×10^6^ AN3CA cells), OE-SLERT (caudal vein injection of 2×10^6^ SLERT-overexpressing AN3CA cells), OE-SLERT+ASO-BDNF (caudal vein injection of 2×10^6^ SLERT-overexpressing AN3CA cells transfected with ASO-BDNF), and OE-SLERT+k252a (caudal vein injection of 2×10^6^ SLERT-overexpressing AN3CA cells and intraperitoneal injection of 25μg/kg k252a). After 4 weeks, the lung tissues were dissected and weighted, followed by H&E staining.

### Statistical analysis

The variations between mean values were determined via one-way (post hoc test) or two-way (Bonferroni post hoc test) ANOVA, as appropriate. ROC curve and Kaplan-Meier plot were used to test the diagnostic and prognostic values of SLERT in EC, respectively. All results are the mean±SD of at least three independent experiments carried out in triplicate. *P* < 0.05 was considered statistically significant.

## Results

### SLERT is located in cell cytoplasm and upregulated in EC

**Fig. 1:**
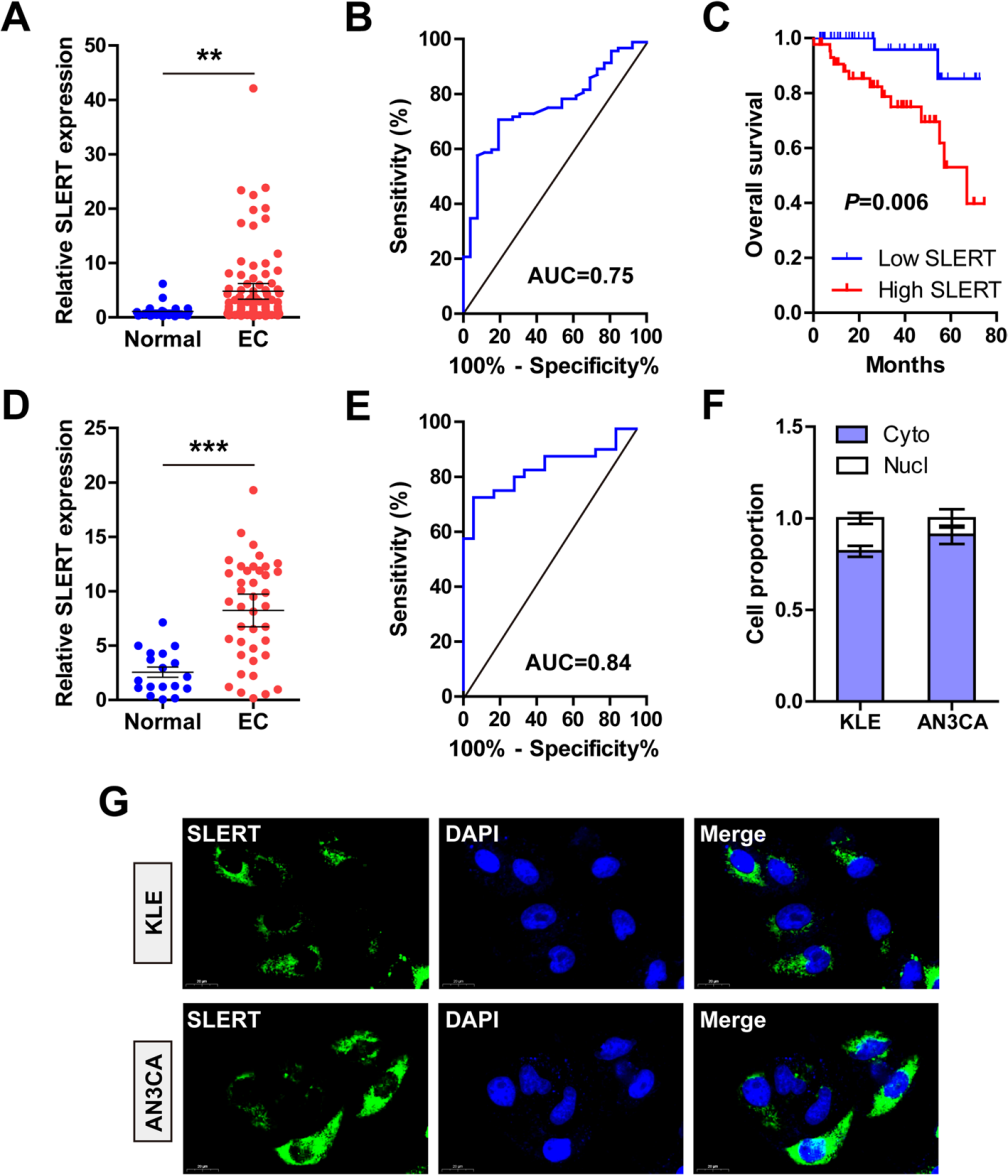
SLERT is highly expressed in EC. **A**, **B** qRT-PCR analysis of SLERT expression in EC and normal tissues, followed by ROC analysis. **C** The survival curve of EC patients with low or high SLERT levels based on median SLERT level. **D**, **E** qRT-PCR analysis of SLERT expression in EC and normal plasma, followed by ROC analysis. **F**, **G** qRT-PCR and FISH testing the location of SLERT in KLE and AN3CA cells. *P<0.05, **P<0.01, ***P<0.001

As shown in Fig. 1A, high SLERT was observed in EC tissues as compared to normal tissues (4.3-fold increase) (*P* = 0.008), with an area under of ROC (AUC) value of 0.75 (95% CI: 0.6604~0.8476) (*P* < 0.001) (Fig. 1B). We analyzed the correlation of SLERT with clinical features; SLERT was significantly correlated with FIGO stage (*P* = 0.002), depth of invasion (*P* < 0.001), lymphovascular invasion (*P* = 0.006), and lymph node metastasis (*P* = 0.018), while it did not correlate with age and histology type (Table 1). In addition, patients with high SLERT had shorter survival time than those with low SLERT (*P* = 0.006) (Fig. 1C). Further, the plasma samples were also collected and tested for SLERT expression. As shown in Fig. 1D, SLERT was notably increased in EC plasma (3.2-fold upregulation) (*P* < 0.001), with an AUC value of 0.84 (95%CI: 0.7384~0.9394) (Fig. 1E). Endogenous SLERT was mainly located in the cytoplasm of KLE and AN3CA cells (Fig. 1F), which was also verified in the visual fluorescence assay (Fig. 1G).

**Table 1:** Association between SLERT expression and clinicopathologic parameters

Parameters	All cases	SLERT expression		*P* value
		Low	High	
Age (years)				
≤ 60	40	22	18	0.4
> 60	52	24	28	
Histology type				
Grade 1+2 endometrioid	67	36	31	0.31
Grade 3 endometrioid	24	10	14	
FIGO stage				
I–II	64	39	25	0.002
III–IV	28	7	21	
Depth of invasion				
≤ 50%	32	24	8	<0.001
> 50%	60	22	38	
Lymphovascular invasion				
Negative	51	32	19	0.006
Positive	41	14	27	
Lymph node metastasis				
Negative	57	34	23	0.018
Positive	35	12	23	

### Knockdown of SLERT attenuates EC cell migration and invasion

**Fig. 2:**
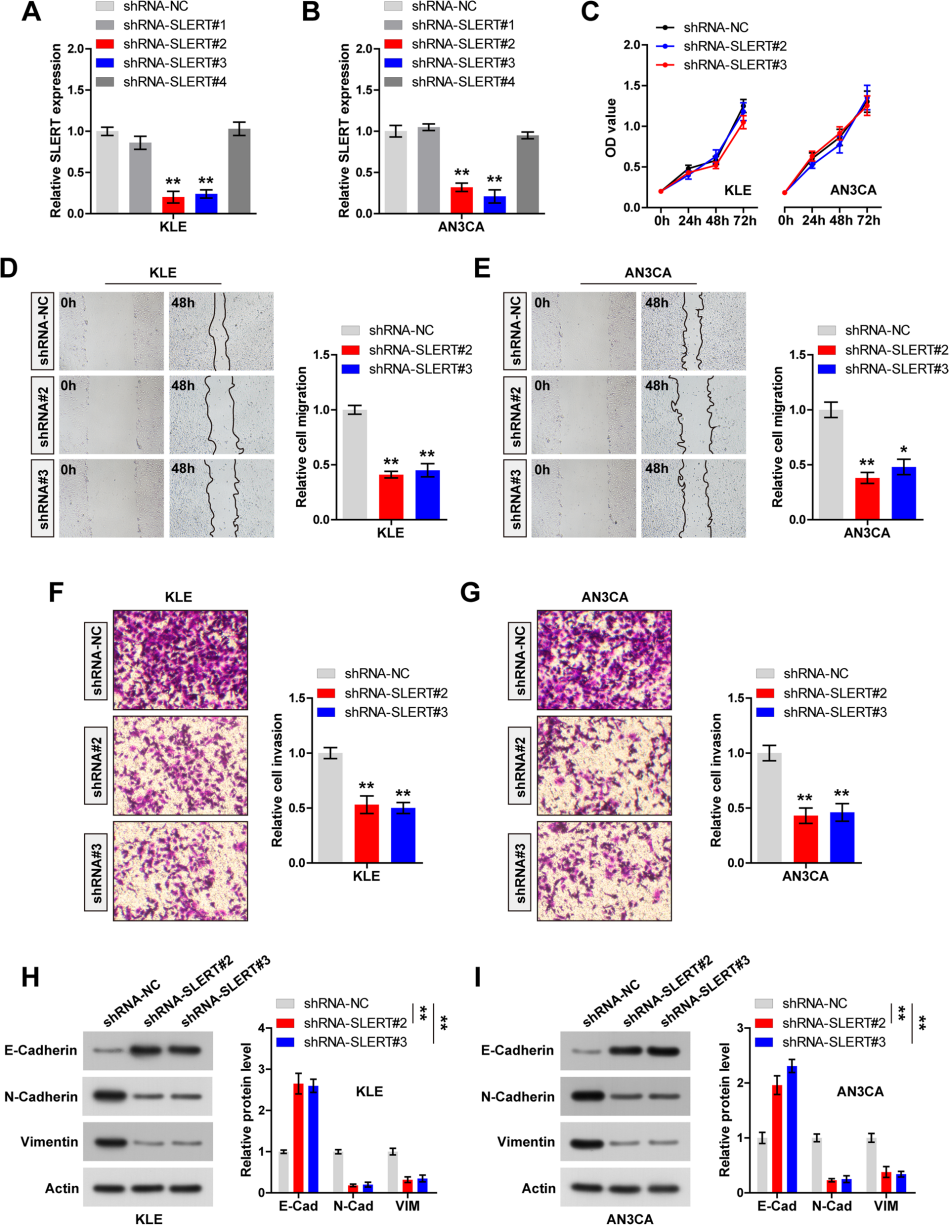
Knockdown of SLERT inhibits EC migration and invasion. **A**, **B** qRT-PCR verifying the knockdown efficiency of these four shRNAs. **C** CCK-8 assay testing cell viability in control and SLERT-silenced KLE and AN3CA cells. **D**–**G** Wound healing and transwell assays testing cell migration and invasion, respectively. **H**, **I** Western blot testing the indicated protein levels after knockdown of SLERT. *P<0.05, **P<0.01

To manipulate SLERT expression in EC cells, we designed four shRNAs targeting SLERT. The results of qRT-PCR showed that shRNA-SLERT#2 and #3 had silence effect (*P* < 0.01) (Fig. 2A, B); we then used them to conduct the functional assays. As shown in Fig. 2C, knockdown of SLERT did not affect cell viability in both KLE and AN3CA cells. However, the distance of cell migration was significantly reduced after silencing of SLERT (*P* < 0.01) (Fig. 2D, E). Besides, the number of SLERT-silenced cells invading matrigel was less than that of control cells (*P* < 0.01) (Fig. 2F, G). Given that epithelial-mesenchymal transition (EMT) is the key phenotype of metastasis, we then tested some EMT markers. As expected, the epithelial marker E-cadherin was remarkably increased, while the mesenchymal markers N-cadherin, Vimentin, and Snail were significantly decreased in SLERT-depleted KLE and AN3CA cells in comparison to control cells (*P* < 0.01) (Fig. 2H, I, Figure S1).

### SLERT increases BDNF mRNA stability in EC cells

**Fig. 3:**
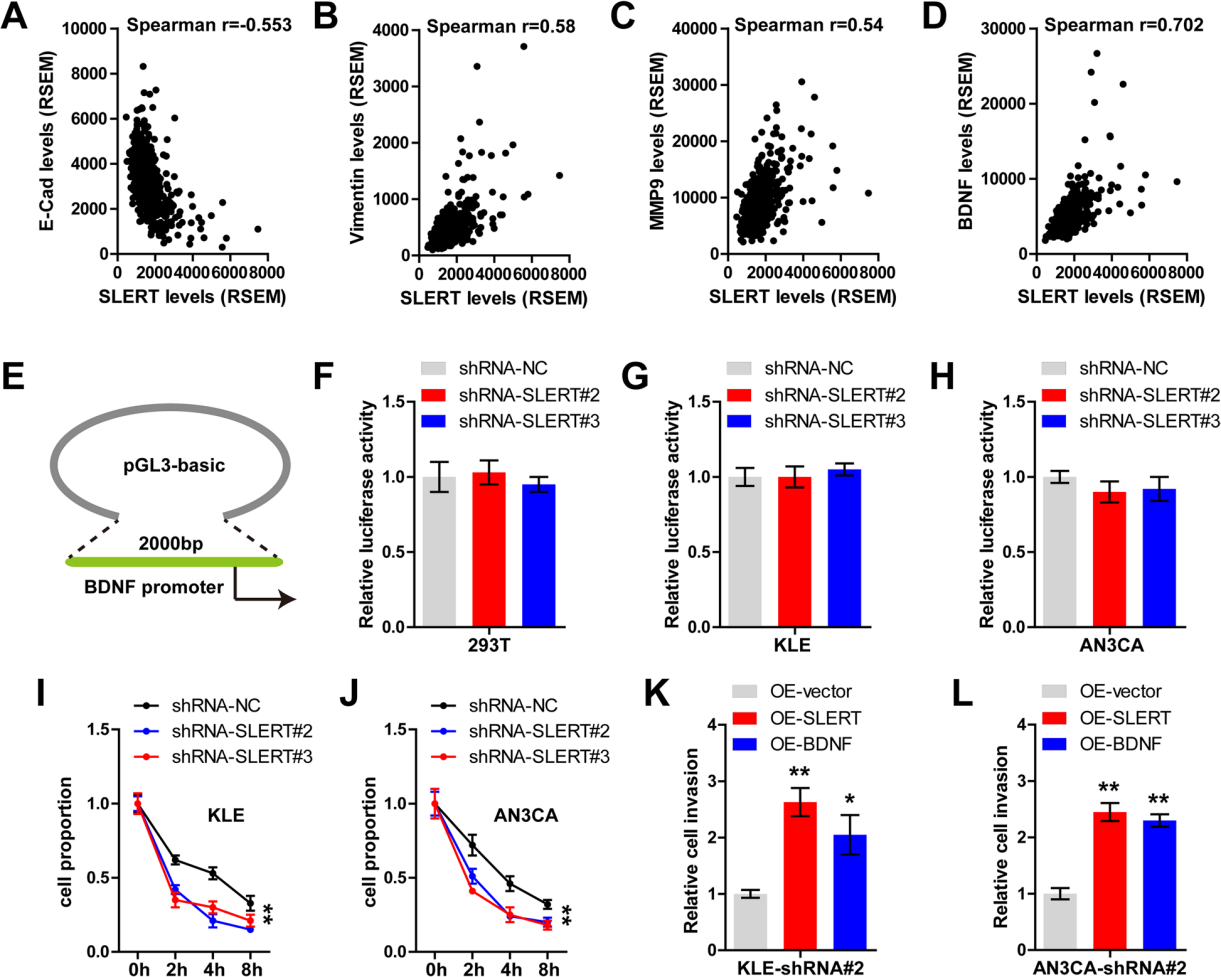
SLERT increases BDNF mRNA expression. **A**–**D** TCGA-EC database showing the correlations between SLERT and E-cad, VIM, MMP9, and BDNF. **E** The cartoon showing the construction of pGL3-basic vector containing BDNF promoter. **F**–**H** Luciferase reporter assay in HEK293T, KLE, and AN3CA cells with SLERT knockdown. **I**, **J** qRT-PCR analysis of BDNF mRNA level in SLERT-silenced KLE and AN3CA cells after treatment with Actinomycin D. **K**, **L** Transwell testing the invasion of SLERT-silenced KLE and AN3CA cells transfected with SLERT or BDNF expression plasmid. *P<0.05, **P<0.01

Through analyzing TCGA-EC database, we found that SLERT was significantly negatively correlated with E-cadherin (*P* < 0.001) (Fig. 3A), whereas positively correlated with Vimentin (Fig. 3B) and MMP9 (*P* < 0.001) (Fig. 3C). Importantly, BDNF, a well-known EMT inducer, was strongly correlated with SLERT expression (*r* = 0.702) (Fig. 3D), hinting that SLERT may control EMT via BDNF. Subsequently, we inserted the promoter of BDNF into pGL3-basic vector and conducted luciferase reporter assay (Fig. 3E). The results showed that knockdown of SLERT had no effect on BDNF promoter activity (Fig. 3F–H). However, after treatment with Actinomycin D, a transcription inhibitor, BDNF mRNA levels were significantly reduced in KLE and AN3CA cells with SLERT knockdown (*P* < 0.01) (Fig. 3I, J), implying that SLERT inhibits BDNF mRNA decay. Functionally, overexpression of SLERT or BDNF drastically enhanced the invasiveness of SLERT-silenced EC cells (*P* < 0.01) (Fig. 3K, L).

### SLERT increases m^6^A level of BDNF mRNA via METTL3/IGF2BP1 axis

**Fig. 4:**
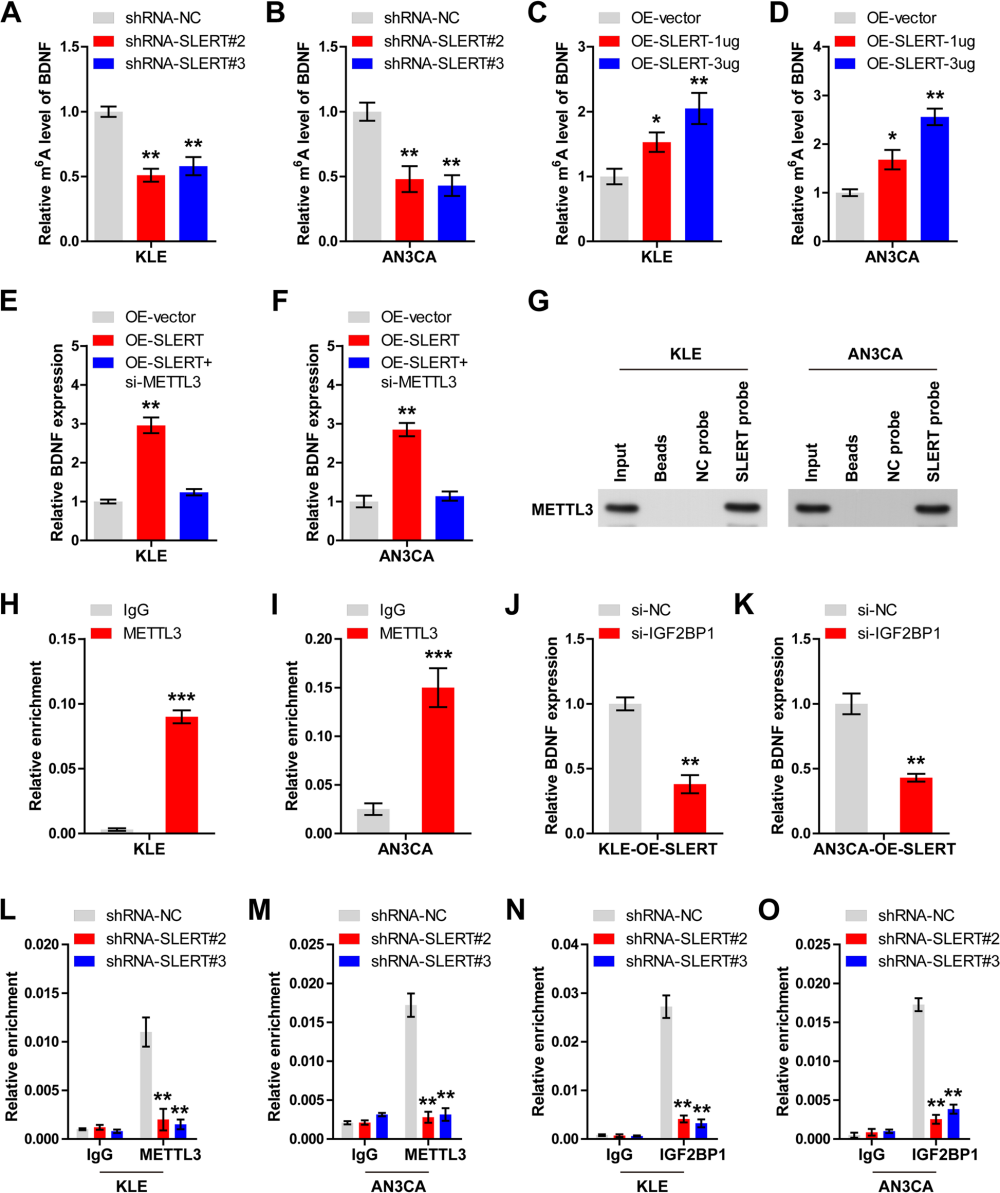
SLERT increases BDNF m^6^A level via METTL3/IGF2BP1 axis. **A**–**D** Detection of BDNF m^6^A levels in KLE and AN3CA cells with SLERT knockdown or overexpression. **E**, **F** qRT-PCR analysis of BDNF mRNA expression in SLERT-overexpressed EC cells transfected with METTL3 siRNA. **G**–**I** RNA pull-down and RIP assays testing the interaction between METTL3 and SLERT in EC cells. **J**, **K** qRT-PCR analysis of BDNF mRNA expression in SLERT-overexpressed EC cells transfected with IGF2BP1 siRNA. **L**–**O** RIP assay testing the enrichment of METTL3 or IGF2BP1 on BDNF mRNA after SLERT silencing. *P<0.05, **P<0.01, ***P<0.001

Given that m^6^A modification is critical for the stability of mRNA [13], we then wondered whether SLERT affected BDNF stability via m^6^A. As expected, the m^6^A levels of BDNF were markedly decreased after SLERT knockdown (*P* < 0.01) (Fig. 4A, B); oppositely, exogenous expression of SLERT increased BDNF m^6^A levels in a dose-dependent manner (Fig. 4C, D). Further, BDNF mRNA expression was increased in SLERT-overexpressed EC cells, and this effect was evidently abolished by silencing of METTL3, a m^6^A “writer” (*P* < 0.01) (Fig. 4E, F). Importantly, RNA pull-down assay showed that METTL3 was massively enriched by SLERT probe (Fig. 4G); similarly, SLERT was also pulled down by METTL3 antibody (*P* < 0.001) (Fig. 4H, I), suggesting the interaction between SLERT and METTL3. IGF2BP1 is a m^6^A “reader” that stabilizes mRNA modified by m^6^A [14]. Silencing of IGF2BP1 reduced the expression of BDNF mRNA in EC cells with SLERT overexpression (*P* < 0.01) (Fig. 4J, K). Moreover, knockdown of SLERT led to less binding of METTL3 (*P* < 0.01) (Fig. 4L, M) and IGF2BP1 to BDNF mRNA (*P* < 0.01) (Fig. 4N, O).

### SLERT induces EMT by regulation of BDNF/TRKB signaling

**Fig. 5:**
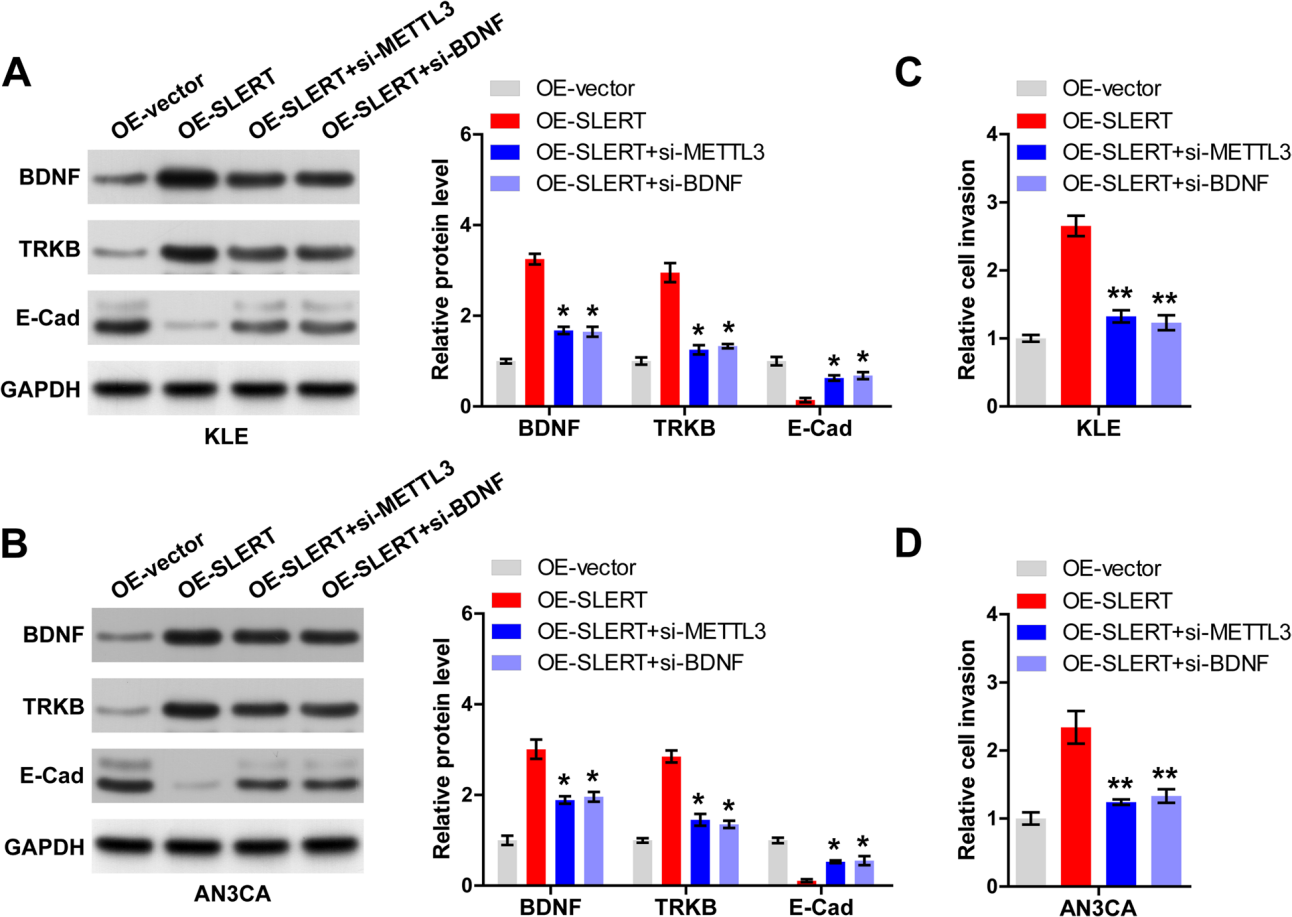
SLERT activates BDNF/TRKB signaling. **A**, **B** Western blot testing BDNF, TRKB and E-caderin protein levels in SLERT-overexpressed KLE and AN3CA cells transfected with METTL3 or BDNF siRNA. **C**, **D** Transwell assay testing the invasion of SLERT-overexpressed KLE and AN3CA cells transfected with METTL3 or BDNF siRNA. *P<0.05, **P<0.01

As shown in Fig. 5A, B, BDNF and its downstream TRKB protein levels were increased, while E-cadherin protein was reduced in KLE and AN3CA cells with SLERT overexpression (*P* < 0.05). However, the above effects were partially abolished by silencing of METTL3 or BDNF (Fig. 5A, B). Functionally, the enhanced cell invasion caused by SLERT overexpression was effectively blocked after METTL3 or BDNF knockdown (*P* < 0.01) (Fig. 5C, D).

### SLERT promotes EC cell metastasis in vivo

**Fig. 6:**
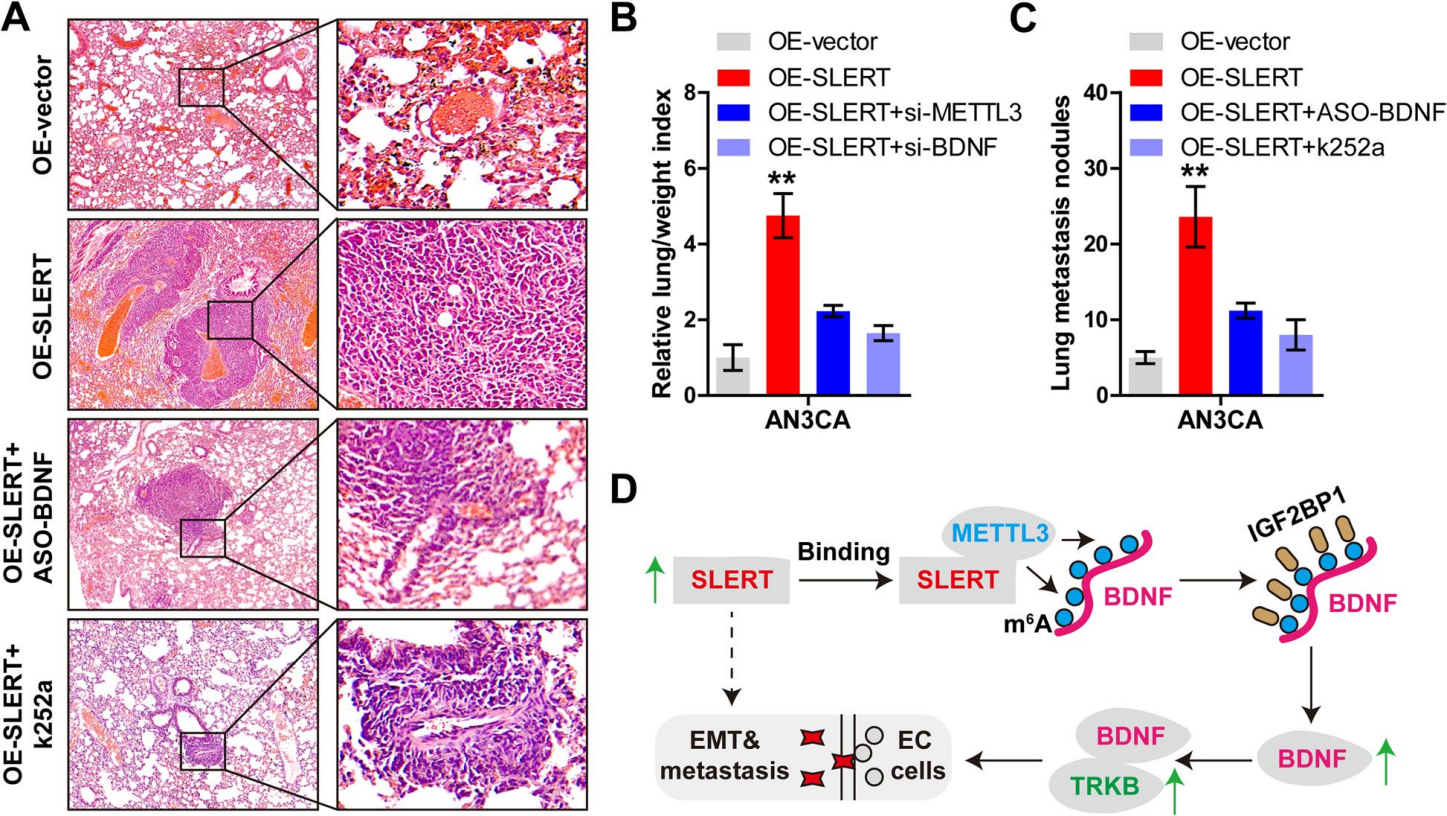
SLERT promotes EC metastasis in vivo. **A**–**C** The representative images/number of lung metastasis nodules and lung/weight index (the number of metastatic nodules divided by body weight) in the indicated four groups (*n* = 5 per group). **D** The proposal model showing the metastasis-promoting role of SLERT in EC via activation of BDNF/TRKB signaling in a m^6^A-dependent manner

Lastly, we established lung metastasis model in vivo by caudal vein injection of AN3CA cells into mice. The results showed that the number of lung metastasis nodule (average number: OE-vector, 5; OE-SLERT: 24; OE-SLERT+ASO-BDNF: 11; OE-SLERT+k252a: 8) and lung/weight index were notably increased in SLERT-overexpressing group (*P* < 0.01) (Figure S2, Fig. 6A–C). However, when BDNF was silenced or treated with k252a (an inhibitor of BDNF/TRKB pathway), the number of lung metastatic nodules was significantly reduced (Fig. 6A–C).

## Discussion

In this study, we found a novel lncRNA associated with EC metastasis. High SLERT was closely linked to aggressive clinical features and poor prognosis. The functional data showed that SLERT promoted EC cell metastasis both in vitro and in vivo, whereas it had no effect on cell viability. In terms of mechanism, SLERT directly bound to METTL3 and facilitated the binding of METTL3 to BDNF mRNA, resulting in the increased m^6^A level of BDNF mRNA, thereby enhancing BDNF stability following IGF2BP1 recognition (Fig. 6D). Thus, our data reveal that SLERT potentiates EC metastasis by activating BDNF/TRKB signaling in a m^6^A-dependent manner. More importantly, we also found that SLERT was significantly overexpressed in EC plasma, the AUC value was 0.84, suggesting plasma SLERT may be a promising diagnostic marker in EC, which needs to be verified in a large sample multi-center study.

The majority (about 90%) of cancer-related deaths are caused by primary tumor cells migrating far from their original site [14]. Metastasis is the process of cancer cells spreading to the whole body, in which cancer cells must first pass over or around neighboring cells [15]. Epithelial-mesenchymal transition (EMT) is the key to the initiation of tumor metastasis, which is a reversible process in which epithelial cells with polarity lose the attachment polarity of basement membrane and the ability of tight connection and adhesion between cells under the action of some factors and are transformed into mesenchymal cells with infiltration and migration ability [16, 17]. Herein, we found that SLERT elevated mesenchymal marker (Vimentin and N-cadherin), while reducing epithelial marker E-cadherin expression, suggesting that SLERT enhances EC cell invasion by inducing EMT process. Interestingly, cell viability was unaffected after manipulation of SLERT expression, indicating that lncRNA plays a specific role in a specific environment, which may be broad or narrow [18]. The BDNF/TRKB signaling pathway is critical in the pathogenesis and development of various neurological diseases, especially epilepsy [19]. Recently, extensive studies have shown that BDNF and TRKB are upregulated in many types of cancer, including EC [20], endowing tumor cells with an aggressive phenotype, such as EMT and chemotherapy resistance [21, 22]. These suggest that the BDNF/TRKB signaling may be involved in many human diseases and has important as well as a wide range of biological effects. Our data showed that SLERT increased BDNF expression, followed by activation of BDNF/TRKB signaling, and knockdown of BDNF or k252a treatment significantly reduced lung metastasis nodules caused by SLERT overexpression. These demonstrate that SLERT promoted EC metastasis through inducing EMT via activation of BDNF/TRKB pathway. Of note, depletion of BDNF or k252a did not completely eliminate the metastasis promoting effect of SLERT, hinting that there are other downstream targets or pathways of SLERT, which need further investigation. Moreover, the reason responsible for SLERT upregulation in EC is still unknown, which may involve some epigenetic regulators or transcription factors, etc.

m^6^A methylation is an important epigenetic modification, which is a dynamic and reversible process regulated by methylase (writer), demethylase (eraser) and reader proteins that recognize m^6^A-modified RNA [23]. It is involved in all aspects of eukaryotic RNA metabolism, including mRNA pre-splicing, 3′-end processing, nuclear output, translation regulation, mRNA decay, and ncRNA processing [24]. The key factors regulating m^6^A are frequently dysregulated in human cancer, linking to malignant phenotype and dismal prognosis [25]. A very recent study tested the expression of m^6^A writers, erasers, and readers in EC by comprehensive immunohistochemical analysis and found that METTL3, METTL14, FTO, HNRNPA2B1, and HNRNPC were significantly highly expressed, which were linked to shortened overall survival in EC [26]. Herein, we found that BDNF mRNA was modified by m^6^A via METTL3, and this process was inhibited after SLERT knockdown, suggesting that SLERT is required for BDNF m^6^A methylation catalyzed by METTL3. Further RNA pull-down and RIP data revealed that SLERT directly bound to METTL3, facilitating the binding of METTL3 to BDNF mRNA. The fate of m^6^A-modified RNA varies according to different readers, including degradation, increased stability, or translation promotion or suppression. IGF2BP1 is a well-documented mRNA stability enhancer via m^6^A recognition [14], for example, IGF2BP1 stabilized PEG10 mRNA in a m^6^A-dependent manner and promoted EC cell proliferation and invasion [27]. Myc proto-oncogene is modified by m^6^A and read by IGF2BP1, resulting in increased mRNA stability and maintaining breast cancer stem cell stemness [28]. In this study, we found that silencing of IGF2BP1 abolished the increased BDNF level caused by SLERT overexpression, and SLERT depletion led to reduced IGF2BP1 binding to BDNF mRNA. The above data suggest that the SLERT/METTL3/IGF2BP1/BDNF axis is critical for EC metastasis.

In sum, our findings uncover that SLERT acts as a pro-metastasis lncRNA in EC by activating BDNF/TRKB signaling in a m^6^A-dependent manner. Moreover, this study provides a potential diagnostic, prognostic marker as well as therapeutic target for patients with metastatic EC patients.

## Supplementary Information

**Additional file 1: Figure S1.** Western blot analysis of Snail levels in KLE and AN3CA cells with SLERT knockdown.

**Additional file 2: Figure S2.** The body weight of the mice in these four groups.

## Data Availability

The data that support the findings of this study are available from the corresponding author upon reasonable request.

## References

[1] Amant F, Moerman P, Neven P, Timmerman D, Van Limbergen E, Vergote I (2005). Endometrial cancer. Lancet..

[2] Sung H, Ferlay J, Siegel RL, Laversanne M, Soerjomataram I, Jemal A (2021). Global Cancer Statistics 2020: GLOBOCAN Estimates of Incidence and Mortality Worldwide for 36 Cancers in 185 Countries. CA Cancer J Clin.

[3] Brooks RA, Fleming GF, Lastra RR, Lee NK, Moroney JW, Son CH (2019). Current recommendations and recent progress in endometrial cancer. CA Cancer J Clin.

[4] Siegel RL, Miller KD, Jemal A (2018). Cancer statistics, 2018. CA Cancer J Clin.

[5] Kopp F, Mendell JT (2018). Functional classification and experimental dissection of long noncoding RNAs. Cell..

[6] Ali T, Grote P. Beyond the RNA-dependent function of LncRNA genes. Elife. 2020:9. 10.7554/eLife.60583 PMC7584451 33095159

[7] Sauvageau M (2019). Diverging RNPs: toward understanding lncRNA-protein interactions and functions. Adv Exp Med Biol.

[8] Xu L, Huan L, Guo T, Wu Y, Liu Y, Wang Q (2020). LncRNA SNHG11 facilitates tumor metastasis by interacting with and stabilizing HIF-1alpha. Oncogene..

[9] Li B, Kang H, Xiao Y, Du Y, Xiao Y, Song G (2022). LncRNA GAL promotes colorectal cancer liver metastasis through stabilizing GLUT1. Oncogene..

[10] Guo M, Zhang X (2022). LncRNA MSTO2P promotes colorectal cancer progression through epigenetically silencing CDKN1A mediated by EZH2. World J Surg Oncol.

[11] Xing YH, Yao RW, Zhang Y, Guo CJ, Jiang S, Xu G (2017). SLERT regulates DDX21 rings associated with Pol I transcription. Cell..

[12] Wu M, Xu G, Han C, Luan PF, Xing YH, Nan F (2021). lncRNA SLERT controls phase separation of FC/DFCs to facilitate Pol I transcription. Science..

[13] Jiang X, Liu B, Nie Z, Duan L, Xiong Q, Jin Z (2021). The role of m6A modification in the biological functions and diseases. Signal Transduct Target Ther.

[14] Huang X, Zhang H, Guo X, Zhu Z, Cai H, Kong X (2018). Insulin-like growth factor 2 mRNA-binding protein 1 (IGF2BP1) in cancer. J Hematol Oncol.

[15] Suhail Y, Cain MP, Vanaja K, Kurywchak PA, Levchenko A, Kalluri R (2019). Systems biology of cancer metastasis. Cell Syst.

[16] Brabletz S, Schuhwerk H, Brabletz T, Stemmler MP (2021). Dynamic EMT: a multi-tool for tumor progression. EMBO J.

[17] Aiello NM, Kang Y (2019). Context-dependent EMT programs in cancer metastasis. J Exp Med.

[18] Statello L, Guo CJ, Chen LL, Huarte M (2021). Gene regulation by long non-coding RNAs and its biological functions. Nat Rev Mol Cell Biol.

[19] Lin TW, Harward SC, Huang YZ, McNamara JO (2020). Targeting BDNF/TrkB pathways for preventing or suppressing epilepsy. Neuropharmacology..

[20] Alonso-Alconada L, Eritja N, Muinelo-Romay L, Barbazan J, Lopez-Lopez R, Matias-Guiu X (2014). ETV5 transcription program links BDNF and promotion of EMT at invasive front of endometrial carcinomas. Carcinogenesis..

[21] Zou W, Hu X, Jiang L (2020). Advances in regulating tumorigenicity and metastasis of cancer through TrkB signaling. Curr Cancer Drug Targets.

[22] Thiele CJ, Li Z, McKee AE (2009). On Trk--the TrkB signal transduction pathway is an increasingly important target in cancer biology. Clin Cancer Res.

[23] Murakami S, Jaffrey SR (2022). Hidden codes in mRNA: control of gene expression by m(6)A. Mol Cell.

[24] Jang KH, Heras CR, Lee G (2022). m(6)A in the signal transduction network. Mol Cell.

[25] He L, Li H, Wu A, Peng Y, Shu G, Yin G (2019). Functions of N6-methyladenosine and its role in cancer. Mol Cancer.

[26] Ralser DJ, Condic M, Klumper N, Ellinger J, Staerk C, Egger EK. Comprehensive immunohistochemical analysis of N6-methyladenosine (m6A) writers, erasers, and readers in endometrial cancer. J Cancer Res Clin Oncol. 2022. 10.1007/s00432-022-04083-1 PMC10129960 35731272

[27] Zhang L, Wan Y, Zhang Z, Jiang Y, Gu Z, Ma X (2021). IGF2BP1 overexpression stabilizes PEG10 mRNA in an m6A-dependent manner and promotes endometrial cancer progression. Theranostics..

[28] Zhu P, He F, Hou Y, Tu G, Li Q, Jin T (2021). A novel hypoxic long noncoding RNA KB-1980E6.3 maintains breast cancer stem cell stemness via interacting with IGF2BP1 to facilitate c-Myc mRNA stability. Oncogene..

