# Supplementary figures and images for: SLERT, as a novel biomarker, orchestrates endometrial cancer metastasis via regulation of BDNF/TRKB signaling
Source: World J Surg Oncol. 2023 Jan 31;21:27. doi: 10.1186/s12957-022-02821-w (PMC9887878; doi:10.1186/s12957-022-02821-w)

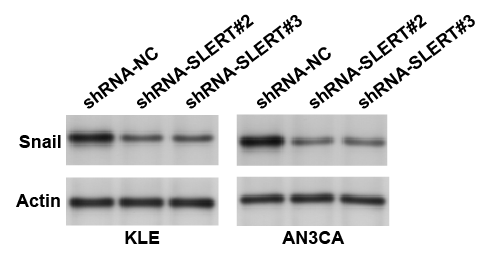

Supplement: Supplementary file 1 — Additional file 1: Figure S1. Western blot analysis of Snail levels in KLE and AN3CA cells with SLERT knockdown. [file 12957_2022_2821_MOESM1_ESM.docx]

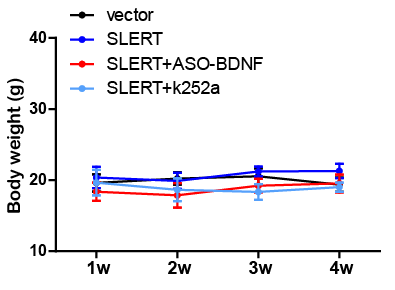

Supplement: Supplementary file 2 — Additional file 2: Figure S2. The body weight of the mice in these four groups. [file 12957_2022_2821_MOESM2_ESM.docx]
